# Radiation in Combination With Targeted Agents and Immunotherapies for Pediatric Central Nervous System Tumors - Progress, Opportunities, and Challenges

**DOI:** 10.3389/fonc.2021.674596

**Published:** 2021-06-30

**Authors:** Bo Qiu, Cassie Kline, Sabine Mueller

**Affiliations:** ^1^ Department of Pediatrics, Division of Pediatric Hematology/Oncology, University of California, San Francisco, San Francisco, CA, United States; ^2^ Department of Pediatrics, University of Pennsylvania Perelman School of Medicine, Philadelphia, PA, United States; ^3^ Division of Oncology, Children's Hospital of Philadelphia, Philadelphia, PA, United States; ^4^ Department of Neurology, University of California, San Francisco, San Francisco, CA, United States; ^5^ Department of Neurosurgery, University of California, San Francisco, San Francisco, CA, United States

**Keywords:** radiation therapies, pediatric brain cancer, brain tumor, Immunotherapy, targeted therapeutic, precision oncology, radiation oncology, combination therapy

## Abstract

Pediatric brain tumors are the most common solid tumors in children and represent a heterogenous group of diagnoses. While some are treatable with current standard of care, relapsed/refractory disease is common and some high-risk diagnoses remain incurable. A growing number of therapy options are under development for treatment of CNS tumors, including targeted therapies that disrupt key tumor promoting processes and immunotherapies that promote anti-tumor immune function. While these therapies hold promise, it is likely that single agent treatments will not be sufficient for most high-risk patients and combination strategies will be necessary. Given the central role for radiotherapy for many pediatric CNS tumors, we review current strategies that combine radiation with targeted therapies or immunotherapies. To promote the ongoing development of rational combination treatments, we highlight 1) mechanistic connections between molecular drivers of tumorigenesis and radiation response, 2) ways in which molecular alterations in tumor cells shape the immune microenvironment, and 3) how radiotherapy affects the host immune system. In addition to discussing strategies to maximize efficacy, we review principles that inform safety of combination therapies.

## Introduction

Collectively, central nervous system (CNS) tumors are the most common solid tumors in children. These tumors represent a heterogenous group of diagnoses ranging from low grade lesions that can be observed or cured through surgical resection to aggressive tumors that are uniformly lethal. Insights into diagnosis and prognosis draw from radiographic and histopathologic features. However, these features tell only part of the story, with molecular alterations greatly impacting diagnosis, prognosis, and therapy decisions in many cases. These molecular features include mutations, copy number variations (CNVs), structural variants (SVs), epigenetic and gene expression changes. Current standard of care for pediatric brain tumors involves molecular profiling of tumor samples to facilitate more precise characterization of this heterogeneous group of tumors, and along with this comes the possibility of treating patients with more individualized regimens. For some, this means tailoring the intensity of treatment through risk stratification. For example, de-intensification of chemotherapy and radiation is being tested in the WNT medulloblastoma subgroup given favorable outcomes with current standard of care, multimodal therapy (NCT 02724579). For high-risk diseases, where standard chemotherapies have historically failed and relapse/refractory disease remains common, an understanding of the specific molecular drivers of malignancy offers the hope of improving outcomes for such patients through a more targeted approach. Ultimately, as new therapies are evaluated, assessment of response in the context of molecular features of a tumor will identify molecular determinants of response.

In the quest to improve outcomes for patients with pediatric brain tumors, the armamentarium has grown to include a spectrum of therapies, including surgery, cytotoxic chemotherapy, radiation, molecularly targeted therapies, and immunotherapies. Radiation has been and continues to be standard of care for many pediatric brain tumors and in some cases, such as diffuse midline gliomas (DMG), is currently the only life-prolonging therapy (1). In contrast, targeted therapy has really only been developed in recent years and refers to agents that directly modify specific cellular processes anticipated to drive cancer. The development of such therapies is driven by an understanding of how genetic alterations in cancer cells promote tumorigenesis and helps form the foundation for precision oncology, where specific agents are chosen for a patient based on the features of their individual cancer. This is in contrast to traditional cytotoxic therapies, which broadly hit rapidly dividing cells indiscriminately (2, 3). In addition, it has been long appreciated that tumor progression is associated with down-regulation of anti-tumor immune responses (4). To this end, immune-based therapies either directly stimulate the immune system or disrupt immunosuppressive pathways to enhance anti-tumor immunity. Both targeted agents and immunotherapies have demonstrated early promise in a number of cancer types and hold promise for the treatment of pediatric CNS tumors; however, the strategies for their application in pediatric neuro-oncology and the acute and long-term side effects of these agents are just beginning to be unraveled. Additionally, although these agents have potential to improve outcomes for the highest risk pediatric brain tumors, single agent therapy is likely to be insufficient for most patients and combination strategies that provide additive or synergistic benefit will likely be necessary.

**Figure 1:**
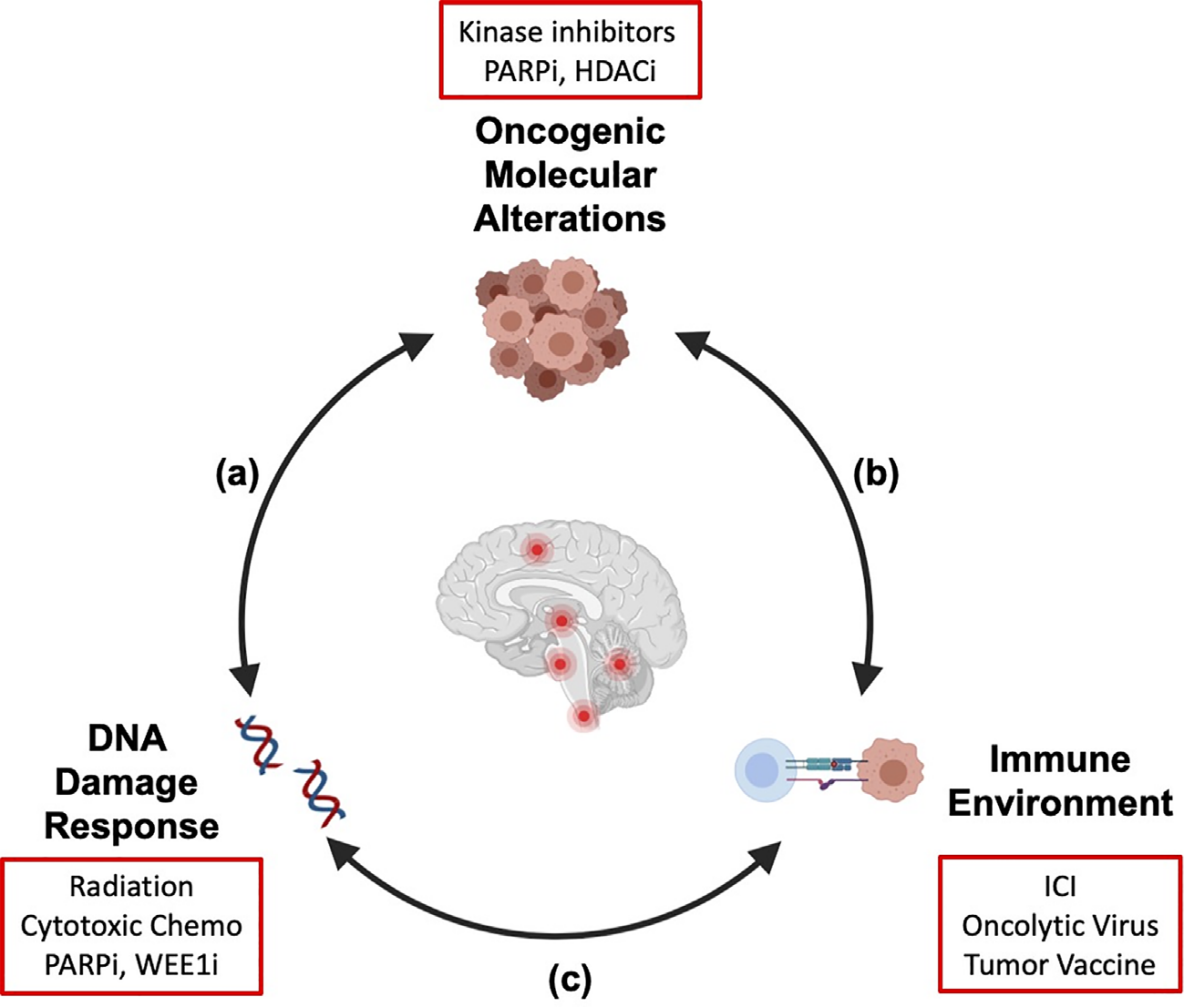
Crosstalk between key hallmarks of cancer informs combination strategies for treatment of pediatric brain tumors. The molecular alterations that drive pediatric brain tumors modulate the cellular response to DNA damage **(A)** and shape the tumor immune microenvironment **(B)**. Radiation therapy induces DNA damage and remodels the tumor immune microenvironment **(C)**. As targeted therapies and immunotherapies are integrated into treatment regimens for patients with pediatric brain tumors, a systematic understanding of these interactions will be necessary to generate combination strategies that are efficacious and safe. Examples of therapeutic agents discussed in this review are shown (red boxes). We propose that his integrated framework should be considered in preclinical and clinical studies to identify molecular determinants of therapy response and inform rational design for combination strategies. Created with Biorender.

We are learning that there is substantial overlap and cross talk between the molecular alterations in brain tumors, the immune microenvironment, and the response to DNA damage by radiation (**Figure 1**). The molecular alterations that drive cellular transformation and cancer cell proliferation also shape the immune environment and the response to exogenous sources of DNA damage like chemotherapy and radiation. In addition, radiation-induced DNA damage and cell death modulate the host immune system, and immune function is necessary for full anti-neoplastic efficacy for radiotherapy (5, 6). It is our hope that a holistic understanding of how these therapies interact will translate into rational therapy combinations and improved outcomes for high-risk pediatric brain cancers in which recurrence is common or cure is unavailable. In this review, we will review mechanistic connections between targeted and immune therapies and radiation that impact efficacy and safety of combining these agents and inform how we move forward with combination strategies. Active clinical trials combining radiation with targeted therapies or immunotherapy for pediatric CNS tumors (at the time of publication of this article) are reviewed (**Table 1**).

**Table 1:** Clinical trials evaluating radiation in combination with targeted therapy or immunot.

Enrollment ongoing or forthcoming					
NCT Number	Phase	Therapeutic Agent	Radiotherapy	Disease Focus	Primary Endpoints
NCT03416530	I	ONC201	Up-front therapy *	H3K27M Gliomas	Dose finding
NCT03690869	I/II	PD1 inhibitor (cemiplimab)	Up-front conventional and hypo-fractionated regimen, re-irradiation	Newly diagnosed DIPG and newly diagnosed and recurrent non-brainstem HGG	Safety and Efficacy
NCT03605550	Ib	BMI1 inhibitor (PTC596)	Up-front therapy	Newly diagnosed DIPG and non-brainstem HGG	Dose finding, Safety
NCT04482933	II	Oncolytic Herpesvirus (G207)	Single dose	Progressive or recurrent supratentorial brain tumor	Efficacy
**Enrollment Completed**					
**NCT Number**	**Phase**	**Therapeutic Agent**	**Radiotherapy**	**Disease Focus**	**Primary Endpoints**
NCT01922076	I	WEE1 inhibitor (adavosertib)	Up-front therapy	Newly diagnosed DIPG	Dose finding, Safety
NCT02502708	I	IDO1 inhibitor (indoximod)	Up-front therapy	Newly diagnosed DIPG	Safety, Efficacy
NCT02457845	I	Oncolytic Herpesvirus (G207)	Single dose	Progressive or recurrent supratentorial brain tumor	Safety
NCT03178032	I	Oncolytic Adenovirus (DNX-2401)	Upfront therapy following single DNX-2401 injection	Newly diagnosed DIPG	Safety

*Up-front therapy is considered standard of care in these diseases and anticipated to follow routine dosing schedules.

## Characteristics of Pediatric CNS Tumors and Early Signals for Targeted and Immune-Based Therapies

Pediatric CNS tumors represent a spectrum of diagnoses, with imaging, histopathology, and molecular features contributing to an integrated diagnosis (7). The World Health Organization (WHO) classification of CNS tumors, last updated in 2016, incorporated many molecular parameters in defining diagnostic entities, and the pending 2020 update is expected to continue this effort (7). This approach ensures accurate diagnosis and prognosis and can facilitate more targeted approaches to therapy. For pediatric brain tumors, broad histopathologic diagnoses include gliomas and embryonal tumors. Given the frequencies of these diagnoses among pediatric neuro-oncology patients, we will largely focus our review on the efforts to target these challenging tumors.

Pediatric low-grade gliomas (LGG) are the most common pediatric CNS tumors overall, and portend a good overall survival (OS) of approximately 90% (8, 9). Some LGG can be cured by surgical resection alone, but when therapy is indicated for non-resectable cases, standard chemotherapy, targeted therapy, and/or radiation (in select cases) are potential therapy options. Interestingly, LGG tends to be driven single molecular alterations, with activation of the RAS/MAPK pathway being the hallmark alteration in pediatric LGG. These alterations include *BRAF*-rearrangements, gain of function *BRAF* mutations, and loss of function mutations in negative regulators of this pathway, such as *NF1* (10, 11). *BRAF* inhibitors and MEK inhibitors have demonstrated efficacy in patients with LGG and prospective trials are ongoing to compare targeted therapies with traditional cytotoxic chemotherapy regimens in the upfront and recurrent settings (12–15). Given the high overall survival rate in pediatric LGG, we emphasize that a key consideration for therapeutic decision making in this group of patients involves optimizing function and minimizing side effects of therapy. These considerations are built into the Children’s Oncology Group (COG) trials comparing cytotoxic chemotherapy (carboplatin, vincristine) with the MEK inhibitor, selumetinib, in newly diagnosed LGG without BRAFV600E mutation (NCT04166409). In addition to tumor response, this study will prospectively follow vision, motor function, neurocognitive function, and quality of life. The upcoming European Society for Paediatric Oncology (SIOP-E) trial LOGGIC (Low Grade Glioma in Children) will also prospectively compare cytotoxic chemotherapy versus targeted therapies, with primary outcomes including visual and adaptive behavioral measures alongside disease control endpoints. Further, there are ongoing academic as well as industry efforts to harmonize long-term follow up of patients treated with new signaling inhibitors to capture the impact these therapies might have on the developing CNS.

In contrast to pediatric LGG, pediatric high-grade glioma (pHGG) carry a dismal prognosis, with the primary life prolonging therapies being surgery and radiation (16–18). However, anatomic location often limits the role of surgery within midline structures, such as DMG. For such patients, radiation is the only life-prolonging therapy to date (19). While pediatric and adult HGG both have poor prognoses, recent integrated molecular profiling efforts have demonstrated that these are biologically distinct entities when it comes to molecular drivers of tumorigenesis. For example, pHGG located in midline structures frequently harbor histone mutations in H3.1 (*HIST1H3B*) and 3.3 (*H3F3A*) that are very rarely reported in adult patients. These mutations include a substitution of lysine amino acid at position 27 with methionine (H3 K27M), which are most frequently found in DMG, and glycine at position 34 with arginine or valine (H3 G34V/R), which are found in hemispheric pHGG. Numerous other differences in the molecular drivers of pediatric HGG are well described and make these tumors distinct when comparing to adult counterparts (11, 20).

Despite the overall poor prognosis of pHGG, targeted therapies and immunotherapies have demonstrated early efficacy for select tumors with specific molecular findings. Infant HGGs include patients diagnosed younger than three years of age and are a group of tumors that may carry a better prognosis than pHGG diagnosed at an older age (21). Multiple molecular analyses of pHGG revealed that infant HGGs enrich for single driver, receptor tyrosine kinase (RTK) fusions such as *ALK, ROS1, and NTRK* (20, 22, 23). Multiple prospective “basket trials” that enrolled patients based on presence of RTK fusions across pediatric and adult solid tumor histologies, including CNS tumors, have demonstrated safety and durable responses (24–27). These results led to the FDA approval for larotrectinib for solid tumor patients with *NTRK* fusions and entrectinib for solid tumor patients with *ROS1* or *NTRK* fusions. Within RTK-fusion positive infant HGG, these alterations are likely oncogenic, as demonstrated by preclinical models of tyrosine kinase inhibitor (TKI) efficacy and case reports of durable responses in patients receiving TKI therapy (20, 22, 23). Prospective studies are underway to evaluate if infantile HGG with such fusion events demonstrate durable response to TKI and if this treatment strategy can be applied to older patients with RTK fusions (NCT04655404, NCT03213704, NCT02576431, NCT02650401). Unfortunately, pHGG affecting older children, including histone mutant cases, are characterized by a combination of molecular alterations that increase the chances that one agent will fail due to resistance or inherent plasticity in oncogenic pathways driving tumor growth (20, 28, 29). In such cases, combinations of drugs and/or radiation offer the potential of increasing therapeutic response and reducing risk of resistance. ACNS1723 is one active phase II clinical trial examining the role for combination maintenance therapy with dabrafenib (BRAFV600E inhibitor) and trametinib (MEK inhibitor) for BRAF V600E mutant pHGG (NCT03919071).

In contrast to the infant HGG, hypermutated HGG that arise in the context of constitutional mismatch repair deficiency (CMMRD) exhibit some of the highest mutation rates in human cancer (30). In the clinical experience with immune checkpoint inhibitors (ICI), tumor mutation burden (TMB) has emerged as a molecular determinant of treatment response and there are now case reports of durable response to ICI in pediatric patients with CMMRD associated hypermutated HGG (30). Unfortunately, the data from the largest prospective trials in newly diagnosed adult HGG, occurring outside the context of CMMRD, revealed no survival difference in patients treated with the ICI nivolumab as maintenance therapy following up front radiation, when compared to bevacizumab, suggesting that single agent ICI was not sufficient to drive a clinically meaningful antitumor immune response in these patients (31). A smaller prospective study in adults with recurrent HGG demonstrated a signal for survival benefit with neo-adjuvant therapy using the ICI pembrolizumab, prior to surgical re-resection, suggesting that timing of immunotherapy may impact the ability to overcome immunosuppressive signals in glioma (32). Collectively, these results demonstrate that single-agent targeted therapies or ICIs may be most effective in specific, rare patients with unique alterations (ie. RTK fusions and hypermutation in setting of CMMRD). For most patients however, the absence of response to single agents illustrates a need to 1) increase our understanding of determinants for response to targeted therapies or immunotherapy and 2) consider combinations of radiation, targeted agents, and immunotherapies (**Figure 1**).

Medulloblastoma is the most common malignant pediatric brain tumor in children and young adults. Standard of care involves multi-modal therapy including maximal safe surgical resection followed by adjuvant chemotherapy and radiation. This regimen is able to cure many patients, but is associated with acute and long-term morbidity due to intensive chemotherapy and craniospinal radiation (33). Recent advances in the molecular profiling of medulloblastoma have revealed distinct biological subgroups with varying pathogenesis and clinical behavior: Wingless (WNT), sonic hedgehog (SHH), group 3, group 4 (33–35). With standard therapy, WNT subgroup patients do quite well, and as a result, clinical trials assessing lower intensity therapies for these patients are under way (NCT 02724579). Relapsed/refractory disease is more common in the remaining subgroups. Accounting for the distinct biology and prognosis of medulloblastoma sub-groups, an open study at St Jude Children’s Research Hospital is exploring risk adapted therapy based on disease staging, sub-group assignment, molecular features, and extent of surgical resection (NCT01878617). Patients with SHH sub-group tumors will also receive vismodegib, a small molecular inhibitor of SHH pathway signaling that targets the G protein coupled receptor Smoothened, with up-front therapy. Phase II data have demonstrated response to vismodegib in a subset of patients with relapsed medulloblastoma and, together with preclinical studies, have shed light on the molecular determinants of response (36–38). In addition, biological sub-types can be identified though integrated molecular analysis with methylation and gene expression profiling, which may further elucidate potential therapeutic targets in these high-risk tumors (39).

Overall, we are beginning to unravel how best to use novel targeted and immune therapies for pediatric brain tumor treatment; however, much work remains on how to best maximize their impact, particularly as part of multi-modal approaches.

## Targeted Therapies and Radiation

### DNA Damage Response Pathways – TP53, WEE1, BRCA, PARP

DNA damage is a key mechanism by which both standard chemotherapy and radiation elicit tumor cell death. We are now beginning to understand that underlying genetic drivers of pediatric brain tumors may function as molecular determinants of radiation response. This understanding may facilitate prognostication for patients receiving radiotherapy, but also provides rationale for targeting cellular processes that drive radio-resistance to enhance response. Radiotherapy induced cell death often occurs in a *TP53*-dependent manner. For example, *TP53* mutant or null DIPG demonstrate radio-resistance, as evidenced by *in vitro* assays from cell lines derived from treatment naïve biopsy specimen and in the more rapid development of disease recurrence following radiation in these patients (40). In some instances, tumor cells upregulate DDR and cell cycle checkpoint machinery to tolerate the genomic insults that arise during cellular transformation, which can promote radiation resistance. An example of this is WEE1, a tyrosine kinase involved in the G2/M checkpoint and overexpressed in pHGGs and high risk medulloblastoma (41, 42). Preclinical data in DIPG has demonstrated that concomitant treatment with WEE1 kinase inhibitor, AZD-1775, impairs radiation-induced G2/M cell cycle checkpoint and enhances radiation-induced cell death in pediatric glioma cell lines. Molecular analyses of primary medulloblastoma have also demonstrated *WEE1* overexpression alongside amplification of the MYC family of protooncogenes (*MYC* or *MYCN)*, which characterize high risk disease in patients from SHH, Group 3, and Group 4 sub-groups. Preclinical data indicate that MYC or MYCN overexpression enhances sensitivity to WEE1 inhibition, possibly due to a vulnerability generated by MYC-induced replication stress (42). A phase I/II study of WEE1 inhibitor, AZD1775 (adavosertib), in combination with irinotecan in relapsed refractory pediatric solid tumors, including CNS tumors, has demonstrated tolerability (ADVL 1321) with mainly hematologic and gastrointestinal dose limiting toxicities that are in line with single agent toxicities (43). The phase II expansion of this study included patients with relapsed, refractory medulloblastoma, though these results have not been reported yet. Concurrent chemo/radiotherapy and WEE1 inhibition in newly diagnosed patients was also recently explored in a phase I study of adavosertib in combination with up front radiotherapy in newly diagnosed DIPG (NCT01922076). Interim evaluations have demonstrated safety of this combination, with ongoing analyses pending (44).

Pharmacologic agents that directly target DDR pathways may be capitalized on as a therapeutic strategy, as the molecular alterations that drive tumorigenesis often alter the cellular response to DNA damage and generate vulnerabilities that are not present in normal, non-transformed cells (45). For example, BRCA mutated cancers that are HR-deficient are vulnerable to inhibition of PARP1-mediated base excision repair and NHEJ – an example of synthetic lethality (46). In addition to breast and ovarian cancer, patients with medulloblastoma and glioma can carry germline BRCA1/2 deficiency, making these tumors potentially vulnerable to therapy with PARP inhibitors (47, 48). While the efficacy of PARP inhibitors were first demonstrated in BRCA-deficient cancers, PARP inhibitors have now proven to be effective in select tumors without BRCA mutations. For example, PARP inhibition sensitizes HGG, medulloblastoma, and ependymoma cell lines to ionizing radiation (49). This suggests that BRCA mutation is not the sole molecular determinant for HR-deficiency or vulnerability to PARP inhibition. Oncogenic mutations in the isocitrate dehydrogenase genes (*IDH1* and *IDH2*), found within various human tumors including HGG, are associated with HR-deficiency and also increase sensitivity to PARP-inhibition in the absence of BRCA-mutation (50, 51). In this setting, the impairment in HR machinery is driven by the oncogenic metabolite 2-hydroxyglutarate – a product of mutant IDH enzymes. Based on these findings, an open study investigating the PARP inhibitor BGB-290 in combination with temozolomide (TMZ) in newly diagnosed or recurrent IDH-mutant HGG, with newly diagnosed patients enrolling after completion of radiation is now enrolling (NCT03749187). Ongoing studies exploring mutation signatures that predict HR-deficiency will hopefully identify a greater number of HR deficient tumors that may benefit from PARP inhibition (52–54).

A series of clinical trials are underway to investigate combination therapies with PARP inhibitors and radiation in pediatric and adult HGG and highlight several principles that are relevant to combination of targeted agents with chemo/radio-therapy. A phase I/II study in newly diagnosed DIPG patients was performed by the Pediatric Brain Tumor Consortium (PBTC) to identify a safe dosing regimen for the CNS-penetrant PARP inhibitor veliparib and determine the safety/efficacy of combination with up front radiotherapy and maintenance TMZ (55). This trial stopped early due to no identified survival benefit compared to historical controls (a common design in pediatric CNS tumor trials due to limited equipoise for side by side comparisons to single-agent strategies) and also poor tolerance of TMZ dose escalation in combination with velapirib. Dose limiting toxicities for the combination were predominantly hematologic, consistent with overlapping toxicities of TMZ and PARP inhibitors. In adult patients, the phase I OPARATIC study in recurrent GBM demonstrated that the PARP inhibitor, olaparib, penetrated to tumor in of 100% of patients on study and identified a safe dosing strategy for intermittent olaparib dosing in combination with continuous TMZ to overcome overlapping hematologic toxicity (56). Currently, a phase I trial is moving this combination up front with radiotherapy in newly diagnosed GBM patients (57). Varied clinical response to PARP inhibitors is impacted by various factors, including: 1) tumor intrinsic features (such as HR-deficiency) and 2) pharmacodynamic properties of distinct PARP inhibitors. Pre-clinical work has demonstrated that anti-tumor activity of PARP inhibitors is not only impacted by inhibition of enzymatic function (suppression of parylation), but also by sequestration (“trapping”) of PARP complexes at sites of DNA damage – preventing efficient repair and leading to cell death (58, 59). PARP trapping potency does not always correlate with extent of enzymatic inhibition and different PARP inhibitors are more or less potent at trapping PARP complexes (58). It is not yet clear which of these activities drives anti-tumor activity of PARP inhibitors in patients, but it is plausible that this may be context/tumor specific.

In addition to combining with chemotherapy and radiation, PARP inhibition in tumor cells can modulate the immune microenvironment through upregulation of tumor cell PD-L1 expression. This upregulation subsequently results in immunosuppressive effects on T cell mediated anti-tumor immunity (60). In this setting, combination of PARP inhibition and anti-PD-L1 therapy improved survival in orthotopic mouse models of high-risk breast cancers. The phase I/II basket trial examining olaparib and the anti-PD-L1 antibody, durvalumab, in patients with germline *BRCA*-mutated metastatic breast cancer (MEDIOLA) demonstrated that this combination therapy was well tolerated with a safety profile similar to individual agents and associated with objective response in 63% of patients (61). This work demonstrates that targeted therapies against tumor cell intrinsic processes exert effects on the tumor microenvironment and highlights thoughtful design of discovery-based combination therapy trials to examine these effects in patients (**Figure 1**).

### MAPK Pathways – BRAF, NF1, PTPN11

A large body of work on MAPK pathway alterations in human cancer has revealed complexities of how this pathway promotes tumor progression (62). Certainly, drugs that inhibit MAPK signaling affect tumor growth by down-regulating mitogenic signals driven by oncogenic alterations in this pathway. Preclinical work indicates that oncogenic MAPK signaling also modulates DDR and response to radiation (**Figure 1**). Dasgupta et al. reported that BRAF inhibitors enhanced radiosensitivity in *BRAF V600E* mutant glioma (63), possibly through disruption of BRAF-mediated upregulation of non-homologous end joining (NHEJ) machinery as seen in radio-resistant papillary thyroid carcinoma (64). Additionally, MAPK signaling in tumor cells elicits cell-extrinsic effects by shaping the tumor microenvironment in ways that can be further exploited therapeutically with immunotherapy combinations. For example, exploratory molecular analysis of patients enrolled in the HERBY phase II study investigating bevacizumab in combination with radiation/temozolomide in pediatric HGG revealed a positive correlation between MAPK pathway activation (alterations in *NF1*, *BRAF*, *PTPN11*, *PTPN12*) and CD8 T cell effector gene expression signature (29). While the net effect of immune signatures on response to immunotherapy is complex, retrospective analyses of adult GBM patients treated with ICI also revealed enrichment of MAPK pathway alterations in responders (65). These data suggest that MAPK-activated high-grade tumors may be more immunogenic and responsive to agents that enhance anti-tumor immune response. Preclinical studies in melanoma have also revealed that combined BRAF and MEK inhibition induces cancer cell death *via* pyroptosis – a highly inflammatory form of programed cell death (66), which triggers an anti-tumor immune response that persists even after drug treatment is completed. Considering these findings in the context of radiation-induced inflammation, there may be opportunities for additive or even synergistic impact when bringing these therapies together in CNS tumors.

### Additional Promising Targeted Therapy and Radiation Combinations in Pediatric CNS Tumors

Activation of the PI3K/AKT pathway and aberrant chromatin regulation are common features of high-risk pediatric brain tumors, including histone mutant DMG, medulloblastoma, and HGG, and may represent additional therapeutic vulnerabilities (20, 39). For instance, the dual histone deacetylase inhibitor (HDAC)/PI3K inhibitor, CUDC-907 (fimepinostat), has enhanced radiation-induced DNA damage and cell death in orthotopic models of HGG and DIPG (67). Building on these findings, a target validation study of fimepinostat in newly diagnosed DIPG, recurrent medulloblastoma, and recurrent HGG is ongoing (NCT03893487). If CNS penetration and safety are demonstrated in this study, prospective studies in combination with up-front radiation for these diagnoses may be the next phase of study for these diseases. Similarly, the polycomb repressive complexes (PRC1 and PRC2), large multimeric protein complexes involved in gene silencing *via* chromatin regulation, are implicated in a variety of human cancers (68). Multiple studies have demonstrated a tumor-promoting function of BMI1, a ubiquitin ligase and PRC1 component, in DIPG (69–71). Preclinical studies demonstrate that inhibition of BMI1 impaired tumor cell proliferation, promoted cell differentiation, and sensitized cells to radiation induced DNA damage (69). Based on these findings, an open phase Ib trial is investigating the BMI1 inhibitor PTC596 in combination with up-front radiation in newly diagnosed DIPG and non-brain stem pHGG (NCT03605550).

ONC201 is another small molecule inhibitor actively undergoing investigation in combination with radiation. The drug is an imipridone compound that was originally identified as an inducer of TNF-related apoptosis-inducing ligand (TRAIL) expression in cancer cell lines (72). Mechanistic studies have indicated that ONC201 upregulates expression of TRAIL and its receptor DR5 through activation of the integrated stress response pathway – an evolutionarily conserved cellular adaptation that mediates 1) response to nutrient deprivation and 2) cell death in the setting of irremediable cellular stresses (73, 74). Through genetic and chemical approaches, multiple groups have identified the mitochondrial enzyme caseinolytic protease P (ClpP) as a direct target of ONC201 and demonstrated that ONC201-mediated ClpP activity is required for anti-tumor activity (75, 76). ONC201-dependent ClpP activity led to degradation of mitochondrial respiratory chain proteins, generation of mitochondrial reactive oxygen species (ROS), and activation of a cytotoxic integrated stress response (75). In pre-clinical studies, ONC201 was found to enhance radiosensitivity in orthotopic mouse models of glioma (77). Radiation therapy also enhances cellular ROS levels, through direct radiolysis of water molecules and through generation of mitochondrial ROS, suggesting a possible mechanism underlying ONC201-dependent radiosensitization (78, 79). Based on early signals for efficacy in recurrent H3K27M mutant glioma, this agent was explored in an expanded access program for pediatric and adult patients with this diagnosis (80). While the patient cohorts are small, long-term objective responses were reported in several patients, furthering the signal of potential efficacy in this high-risk group of patients. A current phase II study for H3K27M positive pediatric HGG, including brain stem glioma, is now open (NCT03416530), and includes arms for maintenance therapy after standard of care radiation, therapy at time of recurrence, and in combination with up-front radiotherapy. These studies will determine if ONC201-dependent radio-sensitization translates into therapeutic benefit in patients.

### Perspectives on Combining Targeted Therapies With Radiation – Safety and Toxicity

As targeted treatments are developed, the safety profile of combination therapies is a major consideration. When approaching the combination of targeted therapies with radiotherapy, a useful framework accounts for both acute and late effects of each mode of treatment, with an eye toward overlapping toxicities. Consideration of overlapping toxicities has guided the development of current standard of care chemo/radiation regimens, and has informed early experience in the combination of multiple targeted therapies for pediatric brain tumors (81). Proactive consideration of anticipated overlapping toxicities of radiation and targeted therapies will be vital in the design of safe and efficacious combination regimens for pediatric brain tumors. In addition, long term sequelae of exposure to targeted therapies in the developing pediatric CNS must be considered, especially if utilized in combination with radiotherapy, where long term adverse effects are already well documented.

The use of agents targeting the RAS/MAPK pathway combined with radiotherapy has some of the most mature data in this realm. Retrospective experience is available for combination BRAF inhibitors and CNS directed radiotherapy for melanoma patients with brain metastases and may inform the use of such combinations in pediatric CNS tumors. The skin is a key organ system where overlapping toxicities of radiation and BRAF inhibitors must be considered. In patients receiving concurrent BRAF inhibitor with whole brain radiotherapy, the incidence of radiodermatitis was significantly greater in patients receiving combined therapy: reported as 44% for combination compared to 8% receiving radiation alone (82). Stereotactic radiosurgery for melanoma brain metastases appeared to have a lower incidence of such skin toxicities, as the anticipated total dose to normal skin would be smaller (82). Importantly, long-term follow-up revealed that although higher in incidence, acute radiodermatitis was reversible in all cases and did not lead to lasting cutaneous side effects. Cutaneous side effects include cutaneous squamous cell carcinoma, which develops in the context of compensatory signaling through wildtype BRAF and MEK in non-melanoma skin cells. As a result, such toxicities are interestingly mitigated by the addition of MEK inhibitor (83). Thus, it remains to be understood if rates of radiodermatitis with BRAF inhibitor plus radiation are improved with addition of MEK inhibitor. At least one report demonstrated that patients with melanoma brain metastases treated with combination BRAF inhibitor and stereotactic radiotherapy experienced greater rates of intra-tumoral hemorrhage when compared to radiotherapy alone (84). A caveat when extrapolating this experience to the treatment of primary CNS tumors is that the pathophysiology of CNS metastasis and hemorrhage risk is very distinct, with rate of spontaneous intracranial hemorrhage in brain metastases occurring at a much higher rate than in primary CNS tumors, especially in melanoma (85). Still, such findings extrapolated from this combination highlight the need for rigorous adverse event monitoring in patients receiving novel combinations of BRAF inhibitors with radiation. The impact of tissue tolerance toward radiotherapy is further highlighted in the experience treating patients with concurrent EGFR inhibitors and radiotherapy. Schwer et al. reported that concurrent gefitinib and stereotactic radiosurgery in fifteen patients with recurrent glioma was well tolerated (86). On the other hand, experience with extra-cranial radiotherapy with EGFR inhibitors for thoracic tumors demonstrated greater incidence of bystander effect like stomatitis and pneumonitis (87). This likely stems from CNS tissue being largely post-mitotic, as opposed to the continuously renewing mucosal and epithelial tissues.

In addition to injury of neighboring non-tumor tissues, acute toxicities of radiotherapy can derive from achieving tumor cell death and activation of host immune response in the tumor. A commonly encountered outcome of this treatment effect is radiographic and clinical pseudoprogression. On-target, anti-tumor response to radiation can be associated with tumor cell death, immune activation, and edema, leading to the phenomenon of pseudoprogression following radiotherapy. With respect to brain tumors, radiographic pseudoprogression is defined as increased contrast enhancement and other signs of tissue edema early following radiation therapy, which ultimately subsides without a change in disease directed therapy (88–90). The latter feature distinguishes pseudoprogression from true disease progression, which is inherently challenging to tease out on imaging analyses alone. Radiographic pseudoprogression can be associated with an increase in clinical symptoms, and in such cases, corticosteroids are often utilized as supportive care. However, due to side effects of corticosteroids, the anti-VEGF agent bevacizumab is being increasingly deployed as a steroid sparing agent for such patients (91). We may find that pseudoprogression becomes more prevalent in the setting of combination targeted therapies with radiation, perhaps as a result of additive or synergistic effects of these strategies. Recognition of this potential acute effect must also be considered when determining clinical trial endpoints and imaging measures of response, such as progression-free survival, which could be impacted by erroneous declaration of disease progression (92).

While the acute effects of combination therapy with targeted drugs and radiation are starting to be elucidated, late neurocognitive, neuroendocrine, and neurovascular complications remain to be discovered. Given their contemporary development, long-term side effects of targeted therapies alone are not yet well understood. As discussed above, active prospective trials evaluating the utility of MEK inhibitor for pediatric LGGs include long-term follow up assessments of vision, motor function, neurocognitive function, and quality of life. This understanding will inform the potential long-term toxicities of combination MEK inhibitors and radiotherapy. While this combination is not considered a strategy for LGG treatment, therapy for higher grade lesions may involve such combinations. When these agents are being combined with radiation, where the same organ systems (i.e. vision) can be negatively impacted by each independent strategy, care must be taken to monitor patients closely. Like the strategy for MEK inhibitors in LGG, we emphasize the importance of long-term tracking of functional outcomes in patients receiving any targeted therapy. Additionally, cranial radiotherapy carries a risk of inducing small and large vessel vasculopathy and increased stroke risk (93, 94). Kinase inhibitors against a variety of molecular targets affect angiogenic/vascular signaling pathways as well, with vasculopathy reported most frequently in patients treated with BCR/ABL inhibitors for CML (95). As such, emphasis should be placed on ongoing neurovascular imaging as a routine part of late effect monitoring for patients receiving combination therapies. As the number of targeted therapies and the patients who receive them grows, it will be important to develop and employ long-term follow-up guidelines for adverse event monitoring, especially when given concurrently with radiotherapy.

## Immunotherapy and Radiation

Cancer immunotherapy refers to treatments that enhance anti-tumor immune function and anti-tumor immune responses, like the response to pathogens, involve a balance of signals that stimulate and restrain immune activation. This balance safeguards against uncontrolled inflammation and autoimmune disorders, but immunosuppressive signals are also co-opted by tumors to escape immune-mediated elimination. Immune-checkpoint signaling restrains T cell function *via* engagement of inhibitory receptors on the T cell surface (including PD-1 and CTLA-4). Ligands for these receptors (including PD-L1 and B7) can be expressed on tumor cells, stroma, or monocytes. ICI therapies function by disrupting these signals (96). In terms of combination strategies, some of the effects of targeted therapies on the immune system were described in the previous section (i.e. PARP inhibitors increasing expression of tumor cell PD-L1). With regard to radiotherapy, the cell-intrinsic effect of radiation on cancer cells is well appreciated, with radiation induced reactive oxygen species eliciting DNA double strand breaks and subsequent cell death or senescence (1). Notably, immune function is also necessary for the anti-neoplastic effect of radiotherapy, including local cytokine production, modulation of tumor associated myeloid cells, cytotoxic T cell infiltration, and enhanced antigen presentation (5, 97, 98). However, radiation-induced inflammation, like other triggers of the immune system, also include inhibitory signals that restrain anti-tumor immune function, including immune checkpoint pathways (99). Thus, there is significant rationale for the combination of immunotherapy with radiation to overcome the immunosuppressive tumor environment.

A goal of immunological therapies is to trigger a local and systemic immune response to eradicate or control the existing tumor. An adaptive immune response also has the potential to promote long term tumor control or prevention of recurrence, even after the patient has completed immunotherapy (100). Preclinical and clinical evidence suggests that radiation can promote such a systemic anti-tumor response (97, 98). This principle is exemplified by the abscopal effect, which corresponds to tumor response at sites of disease beyond the irradiated tumor. Pre-clinical studies have indicated that this effect is at least partly due to systemic anti-tumor immune response following radiation (97, 98). In patients, this is predominantly retrospectively reported and, to date, is unpredictable – likely reflecting variations in antitumor immune status across patients and tumors. ICI and other immunotherapies have the potential to increase the number of patients who might benefit from treatments that trigger a systemic anti-tumor immune response. Within pediatric brain tumors, including DIPG, medulloblastoma, and ependymoma, clinically apparent disease dissemination can be detected on MRI or CSF cytology. However, even in clinically localized DIPG (on MRI and CSF cytology), microscopically disseminated disease is noted in many patients at time of autopsy (101). This dissemination may have occurred following radiotherapy, but there is also the possibility that microscopic disease dissemination occurred before the time of diagnosis, as is the case for microscopic metastatic dissemination for many solid tumors that appear to be localized at diagnosis (102). Focal radiotherapy alone to the primary tumor site thus may not be sufficient to trigger immune surveillance for microscopically disseminated disease. Additional recent work has implicated a hematogenous route of medulloblastoma dissemination, with subsequent re-seeding of the leptomeningeal space (103). The authors identified circulating tumor cells in newly diagnosed medulloblastoma patients, including those with clinically localized disease, and demonstrated that the hematogenous route can contribute to leptomeningeal dissemination in preclinical disease models. In both of these examples, boosting the systemic anti-tumor immune response may improve the potential for eradicating microscopic disease to prevent relapse or progression.

In this section, we review translational and clinical work around immune regulation and immunotherapy in pediatric brain tumors, with an emphasis on strategies to combine such agents with radiation.

### Immune Environment of Pediatric Brain Tumors

Molecular and histologic profiling of pediatric CNS tumor samples and preclinical disease models have shed light on the immune environment of pediatric brain tumors and provided a glimpse at the molecular determinants of the tumor immune environment. An improved understanding of these determinants will inform patient selection for immunotherapies and the development of rational combination strategies to boost response. Previous immunophenotypic profiling across various types of pediatric brain tumors revealed a spectrum of immune compositions, suggesting that mechanisms shaping the immune environment and extent of immunosuppression are likely distinct across different tumor types (104). Thus, the barrier to overcome tumor-induced immune suppression is likely different for distinct tumor types. For HGG, analyses from the HERBY trial suggested that histone mutant DMG were “immune cold,” while MAPK pathway altered pHGG had greater CD8+ effector cell signature (29). For DMG, this “immune cold” transcriptional signature is corroborated by immunohistochemical and flow cytometry based of immune profiling, which demonstrated a very low T cell infiltration (105). These findings suggest that therapeutic agents that enhance cytotoxic T cell function (i.e. ICI) may not be sufficient as single agents and may need to be combined with therapies that enhance cytotoxic T cell infiltration (i.e. radiation). On the other hand, retrospective analyses of adult GBM patients receiving ICI therapy demonstrated that responders to therapy were more likely to exhibit MAPK pathway alterations (106). Given these findings, along with the greater CD8+ effector cell signature noted in MAPK-altered pediatric HGG, these patients may have a lower barrier to overcoming tumor-related immune suppression and may be more amenable to immunotherapies (29). Recent work has also demonstrated that *TP53* mutations, a common feature of many high-risk pediatric brain tumors, impairs anti-tumor immunity through down-regulation of MHC-I in pre-clinical models of medulloblastoma and DIPG (107). These findings serve as initial insight into the intertwined relationship between histologic, molecular, and immune profiles of CNS tumors and potential mechanisms of vulnerability (**Figure 1**).

Another potential determinant of anti-tumor immunity across human cancers is tumor mutational burden (108). One possible mechanism underlying this relationship is increased immunogenicity from tumor-associated neo-antigens generated by somatic mutation (109). The response of hypermutated HGG in the setting of CMMRD to ICI, as described earlier, is a key example of this (30). However, it is also clear that TMB is not the sole determinant of response to ICI for most tumors. Work from Touat et al. suggests that across adult GBM, the path to hypermutation was more likely to impact ICI response, rather than TMB alone (110). The authors identified two primary paths to hypermutation: 1) *de novo* hypermutated gliomas that developed in the setting of CMMRD or DNA polymerase mutations or 2) a much more commonly observed group of acquired mismatch repair deficits following chemotherapy treatment. In the latter group, the mechanism for acquired hypermutation was potentially due to molecular evolution of tumors under selective pressure from TMZ, as acquired mismatch repair deficits arise from this treatment (111). The patients with acquired hypermutation did not exhibit a greater response to ICI than non-hypermutated patients. Single cell sequencing of tumors with acquired hypermutation demonstrated that mutations were sub-clonal, perhaps explaining the absence of a robust boost in anti-tumor immunity following ICI. Contrast this to *de novo* hypermutated glioma, in which mutations (and neoantigens) were more likely to be truncal and subsequently trigger anti-tumor cytotoxic T cells upon ICI therapy. Furthermore, recent preclinical studies in mismatch repair (MMR) deficient tumors report that TMB, and the presumed associated increase in tumor associated antigens, is not sufficient to elicit anti-tumor immunity or predict response to ICI (112, 113). They demonstrate that sensing of cytosolic DNA, which is increased in MMR deficient cells, *via* the c-GAS-STING pathway is necessary for anti-tumor immune response and response to ICI in preclinical tumor models. Furthermore, they demonstrate that in patients with MMR deficient tumors, downregulation of cGAS-STING is associated with a poor prognosis.

Overall, these studies demonstrate that 1) an understanding of the molecular determinants of anti-tumor immunity can help identify patients that may respond to immunotherapy, and 2) combination therapy will likely be indicated to boost anti-tumor immunity or overcome mechanisms of resistance for most patients. In this section, we review various immunotherapies that can be combined with radiation and mechanisms underlying radiation-induced signaling changes within tumor cells and in the microenvironment that provide rationale for such combinations. In addition to highlighting the potential combinations that maximize efficacy, we discuss key factors that will impact the safety of such combinations.

### Systemic Immunotherapies

Experience with immunotherapies for pediatric CNS tumors is an actively evolving field and as such, experiences with immunotherapy in adult malignancies and other pediatric cancers are pertinent. The CheckMate 143 trial was the first randomized phase III study to evaluate checkpoint blockade in patients with primary brain tumors. This study compared anti-PD-1 inhibition using nivolumab to bevacizumab in adult recurrent GBMs after standard of care surgery and radiation. This study did not find an OS difference in these two groups, but did demonstrate a side effect profile for nivolumab that was similar to those reported when used in other adult malignancies (31). Checkmate 143 included exploratory cohorts that tested combination nivolumab with up front chemo/radiotherapy (114). Preliminary analyses demonstrated that this therapy was well tolerated and prompted ongoing randomized phase III studies examining nivolumab with concurrent chemoradiotherapy in newly diagnosed GBM (Checkmate 498, Checkmate 548). CheckMate 143 also evaluated potential clinical variables that modulate the impact of immune-based therapies. Baseline corticosteroid use is a documented prognostic indicator in patients with GBM (115) and corticosteroid use carries the risk of impairing lymphocyte function. In multi-variate analyses in CheckMate 143, patients with no baseline steroid use had a significantly greater OS when treated with nivolumab, when compared to those on steroids. No significant difference was observed in the bevacizumab group. While potentially subject to confounding factors, this trend suggested the possibility that steroid therapy impaired therapeutic efficacy in the nivolumab cohort. The impact of corticosteroids on lymphocyte count and function is well documented, and may also contribute to immunosuppressive tumor microenvironment in brain tumor patients (116). As such, the use of bevacizumab as a steroid-sparing agent in treatment of pseudoprogression in patients receiving combination radiotherapy and ICI is commonly considered. The safety of bevacizumab plus ICI is demonstrated in other solid tumor therapies and is being actively explored in a phase II study of GBM (NCT03452579) (117, 118).

ICI use in pediatric oncology has ranged from disease-specific application to basket trials across pediatric solid and CNS tumors (NCT02304458). An initial study using the PD-1 inhibitor, pembrolizumab, in progressive DIPG, enrolled only 5 patients before being put on hold due to neurologic deterioration that appeared to be more rapid than historical controls and cautioned enrollment of subjects with late stage recurrent disease in future immunotherapy trials (119). This study has since re-opened and is continuing to enroll across a variety of brain tumor subtypes, outside of DIPG. Since this report, retrospective and prospective experience has demonstrated the safety of ICI in pediatric CNS tumors, including DIPG, at diagnosis and in the setting of recurrence. Single, retrospective institutional experience with nivolumab combined with reirradiation for recurrent DIPG demonstrated overall tolerability of this combination treatment with some potential signal of benefit with the combination approach (OS 22.9 months with nivolumab and reirradiation *vs.* 20.4 months with reirradiation alone) (120). While the small sample size of this retrospective study limited the ability to form conclusions on efficacy, patients on corticosteroids at the start of combination radiotherapy and nivolumab were all able to wean steroids following treatment, providing some signal of therapeutic benefit.

An ongoing prospective efficacy study of the anti-PD-1 agent, REGN210 (cemiplimab), is currently evaluating the combination with radiotherapy in newly diagnosed patients with DIPG and non-brain stem HGG, as well as with re-irradiation in recurrent HGG (NCT03690869). This study is investigating alternative radiation fractionation strategies in combination with ICI. While a discussion around conventional *vs* hypofractionated therapy is outside the scope of this review, we highlight that alternate fractionated strategies are being investigated in combination studies with immunotherapy, based on clinical and preclinical studies showing that alternate radiation strategies have distinct effects on the tumor immune environment and that sub-ablative radiation doses may have immune-priming effects (121–123). Additional benefits of shorter radiation courses include reduced strain on patients and their families, who often need to travel or relocate to medical centers where radiotherapy is provided, and decreased need for daily anesthesia for the youngest patients. To this end, a matched cohort study investigated safety and efficacy of two hypofractionated strategies for newly diagnosed DIPG patients: 39 Gy in 16 fractions or 44.8 Gy in 11 fractions (124). In this study with 27 children, both regimens were well tolerated and OS and progression free survival outcomes were not statistically different from a matched historical cohort (54 Gy in 30 fractions). In addition to exploring how these strategies affect efficacy, it will be necessary to prospectively identify the safety profile of combination strategies as well. The cemiplimab study is not designed to directly compare the two arms of standard *vs.* hypofrationated radiation, but will provide valuable information on radiation schedules in combination with ICI.

The timing of immunotherapy may also impact efficacy, as neo-adjuvant pembrolizumab, followed by maintenance therapy, significantly extended OS compared to maintenance therapy alone in patients with GBM (32). This suggests that timing of immunotherapy prior to local control measures (surgery or radiation) may boost anti-tumor immune response, perhaps due to inflammatory signaling elicited by local control treatments. To explore this, a randomized double blind, pilot trial of neoadjuvant checkpoint inhibition in recurrent pediatric or young adult HGG is active (NCT04323046). In this study, patients who are undergoing debulking or re-resection as part of their standard care will receive nivolumab, ipilimumab, or nivolumab plus ipilimumab prior to resection. Through the assessment of CNS tumor tissue following neoadjuvant ICI, this study will also augment our understanding of the impact of neo-adjuvant PD-1 inhibition on the immune microenvironment, provide biomarkers of response *vs* resistance, and shed light on future combination strategies to augment PD-1 blockade. Insight gained from this trial may inform investigations of neo-adjuvant ICI in newly diagnosed patients with high-risk brain tumors.

Another immunosuppressive pathway implicated in tumor biology involves the enzyme, indoleamine 2,3 dioxygenase 1 (IDO1). IDO1 is expressed in tumor infiltrating T cells and its enzymatic activity converts tryptophan to kynurenine – a molecule which reduces cytotoxic T and NK cell activity, while promoting the expansion of immunosuppressive regulatory T cell and myeloid derived suppressor cell populations (125). Tumor infiltrating lymphocytes in GBM upregulate IDO1 and greater IDO1 gene expression correlates with worse prognosis in GBM patients (126). This immunosuppressive pathway is active in a number of advanced solid tumors, which led to the development of IDO1 inhibitors. CNS penetrant IDO1 inhibitors have been investigated and demonstrated efficacy in preclinical GBM models when added to radiation and PD-1 blockade (127). Within pediatric brain tumors, a phase I study of the oral IDO1 inhibitor, indoximod, identified a R2PD dose in pediatric patients with progressive high grade brain tumors and demonstrated safety when given concurrently with radiotherapy and temozolomide in newly diagnosed DIPG (NCT02502708) (128). Compared to historical controls, thirteen newly diagnosed patients reported in this study demonstrated median OS of 14.5 months, which is greater than that of historical controls where survival tends to range from 9-12 months, suggesting potential promise of this approach (129, 130). However, biopsy was not a requirement on this study and the absence of prognostically relevant information about tumor biology limits the assessment of treatment efficacy in this small cohort.

Preclinical work has also demonstrated that various pediatric glioma associated antigens can elicit anti-tumor immune responses, which may reflect a novel therapeutic opportunity. Building on these results, a clinical trial assessed sub-cutaneous vaccination with glioma associated antigens (IL-23Ralpha2, EphA2, and survivin) concurrent with up-front radiation or chemo/radiotherapy in newly diagnosed pediatric brain stem glioma and HGG subjects (131). The primary endpoints of this study were safety and assessment for systemic immune response to vaccination. Results demonstrated toleratability and antigen-specific interferon responses in peripheral blood mononuclear cells in the majority of patients. Subjects with DIPG who had evidence of pseudoprogression (4 out of 5) had improved OS compared to those without (median OS 19 versus 11 months). A potential explanation for this is that symptomatic pseudoprogression is associated with a more robust anti-tumor response. Chheda et al. has also demonstrated that H3K27M-specific T cells could be propagated after *in vitro* antigen exposure and that transfer of H3K27M specific T cells led to anti-tumor activity in mouse glioma xenografts, providing a neoantigen target (132). A a phase I/II multi-institutional study is evaluating the combination of a peptide vaccine against H3K27M, alone or in combination with nivolumab, in newly diagnosed patients with H3K27M positive DIPG or DMG (NCT02960230), beginning at 2-8 weeks post initial radiotherapy. In this study, the single agent peptide vaccine was overall well tolerated, with grade 1-2 injection sites reactions being most common (133). Longitudinal immunophenotypic profiling yielded biological correlates to response, including evidence of sustained H3K27M reactive CD8 T cells (39% of patients). Conversely, patients who received dexamethasone therapy, either before or after vaccination, exhibited declining H3K27M specific CD8 cell counts on longitudinal observation and poorer OS. Although steroid dependence is independently associated with worse survival in DIPG patients (134), we highlight again that in the context of immunotherapy, immunosuppressive effects of corticosteroids are likely to impact treatment efficacy and bevacizumab should be considered as a steroid sparing supportive medication in patients receiving immunotherapy (116). Lastly, the combination of tumor vaccine with radiation and/or ICI has the potential to promote a more robust anti-tumor immune response and overcome tumor-related immunosuppression in patients who are immunologic-non-responders to single agent peptide vaccine.

Cytokine release instigated from ionizing radiation induces a local inflammatory environment that further shapes local immune response. The use of exogenous cytokine therapy as an immune adjuvant in combination with radiotherapy has been explored for various malignancies (135). Specifically, TNF-dependent regulation of pathogen or cancer associated immune responses has been the subject of long standing research (136). Preclinical work has identified that combined TNF and immune checkpoint blockade is sufficient to overcome the immunosuppressive tumor microenvironment in two high risk pediatric brain tumors – *TP53-*mutant SHH medulloblastoma and DIPG. A recurrent mechanism of tumor immune escape is down-regulation of surface MHC-I, which is necessary for presentation of tumor associated antigens (TAAs). The authors of this study demonstrated that mutant *TP53* was sufficient to drive immune escape in mouse models of medulloblastoma and DIPG and that this immunosuppression was dependent on *TP53*-mediated down-regulation of MHC-I. In orthotopic tumor models, systemic TNF alpha was sufficient to restore MHC-I expression on tumor cells and enhance response to ICI in mouse models, leading to tumor regression that was associated with lasting systemic anti-tumor immunity that prevented engraftment on repeated tumor challenge. This work demonstrates that *TP53* alterations may serve as key biomarker for tumor-related immunosuppression when compared to *TP53* wildtype counterparts and may require combination strategies. Previous clinical studies with systemic single-agent TNF alpha demonstrated significant dose-limiting acute, systemic toxicities due to inflammatory signaling (137, 138). Notably, in the work summarized above, low doses of TNF that were tolerable for weeks were sufficient to upregulate tumor cell MHC-I and enhance ICI efficacy. These findings suggest that low dose TNF alpha plus ICI may be a viable therapy option in patients with *TP53* mutated brain tumors and highlights the principle that synergistic anti-tumor effects of combination therapies might be obtainable with lower doses than those identified in studies with single agent treatments (i.e. below the maximum tolerated dose for single agents). This strategy is going to be tested in an upcoming trial combining TNF and nivolumab. Notably, radiotherapy is also sufficient to increase local TNF and enhance tumor MHC-I expression, possibly with less systemic toxicity than systemic exogenous TNF (139). It will be of interest to determine if radiation plus ICI would provide similar effects in high risk *TP53* mutant tumors.

### Local Immunotherapies

To overcome tumor associated immunosuppression, local agents have been utilized to stimulate the immune system. Advantages for local immunotherapy deliver include: direct inoculation to overcome the blood brain barrier and limiting systemic toxicities through local injection. An example of this is intra-tumoral injection of unmethylated cytosine-guanosine motifs (CpG-ODN), which are not present in mammalian cells, but correspond to a pathogen associated molecular pattern (PAMP) found in bacterial and viral genetic material (140). Immune responses to CpG are mediated by Toll-Like Receptor 9 (TLR9), which is located primarily on antigen presenting cells, including dendritic cells and CNS microglia, as well as glioma cells (141). Engagement of TLR9 results in inflammatory cytokine production and enhances antigen presentation to CD8 T cells. A phase II randomized study combined CpG injection into the tumor bed at the time of up-front resection in newly diagnosed GBM (142). This therapy was found to be safe but did not enhance survival in this study. Side effects included greater risk of post-operative fever and injection site hematoma, but no severe or lasting adverse events were noted. The combination of CpG with other modalities that enhance immune activation, including radiation and ICI, is now being evaluated (140). Rodent glioma models demonstrated a survival benefit of combined CpG and XRT, an effect that required T cell function (143). These results suggest that local CpG plus radiotherapy provide additive or synergistic benefit to overcoming tumor-induced immune suppression. In high risk pediatric tumors, intra-tumoral delivery of local therapies is employed in various settings, including oncolytic virus injection (discussed in next section) and in novel catheter-based infusion strategies that deliver anti-neoplastic agents *via* convection enhanced delivery (144, 145). Thus, the clinical systems for delivery of local immune-adjuvants are in place and can be explored as another strategy for overcoming tumor associated immunosuppression, in combination with radiation or systemic therapies.

Oncolytic viruses are another avenue for local immunotherapy. These viruses exert anti-tumor activity through several possible mechanisms: direct tumor cell killing, increased tumor associated antigen presentation, and stimulation of a local pro-inflammatory environment. Multiple oncolytic viruses with tropism for CNS tumors have reached the clinic and have demonstrated safety when injected locally as a single agent. For example, oncolytic herpes simplex viruses (oHSV) have a natural tropism for neural tissue and have been modified to restrain viral replication in normal neural tissue while permitting replication in tumor cells (146). In preclinical models, single doses of radiation enhanced oHSV replication and viral-associated tumor cell killing (147). In addition, oncolytic viruses have demonstrated radio-sensitizing effects, with a potential mechanism involving viral-mediated impairment of DNA repair pathways (148). These findings led to combination oHSV and radiotherapy in glioma patients. Intra-tumoral injection of the oHSV G207 with 5Gy single dose focal irradiation has now been found to be safe and result in stable disease or partial reponse in a cohort of nine adult patients with HGG (149). A phase I study investigating the safety profile of delivering oHSV *via* surgically implanted catheters in 12 pediatric patients with recurrent or refractory supratentorial pHGG found that oHSV (10^7^ or 10^8^ plaque forming units, alone or in combination with a single 5 Gy dose of focal radiotherapy) was well tolerated and resulted in no identified peripheral blood virus shedding (150). The authors reported a median OS of 12.2 months (95% CI 8 to 16.4), which is longer than the median OS of 5.6 months in historical cohorts. This study also highlighted the challenges of post-therapy clinical and imaging follow up after local immunotherapy for pediatric CNS tumor therapy. Several patients underwent repeat tissue biopsy per standard care (due to indeterminant MRI findings or new onset neurologic symptoms), facilitating histologic assessment of local immune response to therapy. Immunohistochemistry revealed presence of tumor infiltrating lymphocytes, suggesting that oHSV therapy may help overcome the “immune cold” nature of pHGG. Lastly, the authors found that HSV serologies may serve as a biomarker for G207 therapy benefit, with inferior OS in patients who were HSV seropositive at baseline (median OS 5.1 months) and improved OS in patients who seroconverted during therapy (median OS 18.3 months). A forthcoming phase II trial will assess efficacy of oHSV (10^8^ plaque forming units with 5 Gy radiation) in a larger cohort of relapsed or refractory supratentorial pHGG and provide additional prospective information on determinant of immune and tumor response to therapy (NCT04482933).

As oHSV trials proceed, preclinical efforts are underway to identify combination therapies with oHSV to promote anti-tumor immune response, including concomitant ICI or exogenous expression of pro-inflammatory cytokines *via* the modified oHSV. In a mouse glioma model, combination of oHSV with PD-1 and CTLA-4 blockade led to tumor regression in most mice and prevented tumor engraftment on tumor re-challenge in mice with initial tumor regression, suggesting that this combination therapy generated a lasting anti-tumor immune response (151). In advanced melanoma patients, a randomized phase II reported improved objective response rate to 39% from 18% when oHSV engineered to express GM-CSF was added to anti-CTLA4 therapy (odds ratio, 2.9; 95% CI, 1.5 to 5.5; P = .002) (152). Interestingly, responses were not limited to the injection site (i.e. abscopal effect), suggesting that a systemic anti-tumor immune response was elicited. The most common side effects were very similar to those seen with each single agent.

Adenovirus, poliovirus and measles virus are additional oncolytic viral therapies being studied in the context of pediatric and adult brain tumors. In adults with recurrent GBM, A phase I dose escalation study of single intratumoral injection of DNX-2401, a modified oncolytic adenovirus, found no dose limiting toxicities and noted objective responses in the majority of patients in this cohort (153). Another subset of patients underwent planned tumor re-resection fourteen days following adenovirus injection. Pathological assessments of resected tumors demonstrated immunohistochemical markers of active viral replication and CD8 T cell infiltration. Compared to baseline tissue samples, post-DNX-2401 injection specimens exhibited upregulation of the co-inhibitory TIM3 protein in T cells, but no change in PD-1, PD-L1, or IDO-1 expression. This trial highlights potential benefit of neo-adjuvant immunotherapy prior to planned standard of care re-resection. Such investigational approaches provide valuable assessment of *in vivo* responses to immunotherapy, which helps evaluate the accuracy of pre-clinical models and provide hypothesis generating information to inform future investigations. Another single institution pediatric trial for DNX-2401 in newly diagnosed DIPG patients is currently evaluating the safety of a single virus injection after biopsy and preceding standard of care radiotherapy (NCT03178032). In this study, radiotherapy is initiated three to four weeks following DNX 2401 injection. An interval report describing the first eight patients on study reported no evidence of dose limiting toxicities and indicated that patients were able to discharge from the hospital three to four days post-injection (154). Based on the data from DNX-2401 in adult GBM, it is expected that actively replicating virus should be present in the DIPG tumors at the initiation of radiotherapy and that the immune-stimulating effects of the virus and radiation therapy were active concurrently in these patients.

Poliovirus is another virotherapy, which demonstrates tropism for surface CD155, a marker expressed on many solid tumors including glioma and on antigen presenting cells (APCs) (155). Preclinical data demonstrated that anti-tumor immune activity was driven by direct tumor cytotoxicity and by APC-dependent cytokine release, local inflammation, and T cell stimulation (156). A phase I study with a dose expansion phase treated 61 adult patients with recurrent WHO grade IV glioma with intra-tumoral attenuated poliovirus, delivered by catheter-based convection enhanced delivery (155). Therapy was generally well tolerated and no cases of disseminated encephalitis or meningitis were identified. A dose limiting toxicity was observed in one patient who experienced an intratumoral hemorrhage that the authors attribute to the catheter procedure, rather than local inflammatory effects of the virus. Median OS for study patients was not significantly different from a historical control cohort. However, OS did reach a plateau of 21% in study patients at 24 months, which was sustained at 36 months. While duration of follow-up limited statistical analyses at the time of the report, the historical control group did not exhibit this pattern of sustained OS at these time points. The biological determinants underlying the response in these patients is not understood. An early phase trial for CED-based delivery of this attenuated poliovirus in pediatric patients with recurrent HGG (WHO grade III and IV) is active (NCT03043391). If this therapy is found to be well tolerated, follow-up studies may consider combination therapy with radiation to enhance anti-tumor immunity and overcome tumor-related immunosuppression.

An attenuated measles virus is also under clinical investigation for children and young adults with recurrent medulloblastoma or atypical teratoid rhabdoid tumor (ATRT), which express the CD46 surface marker that mediate measles virus entry (NCT02962167). This trial employs local injection of virus at the time of planned surgical resection for localized recurrence or injection into the subarachnoid space *via* lumbar puncture for patients with disseminated disease at relapse. Both approaches have demonstrated safety and efficacy in preclinical models (157, 158). A study investigating local injection of modified measles virus in adult patients with recurrent GBM has completed enrollment and is pending analysis (NCT00390299). While patients with medulloblastoma and ATRT generally receive craniospinal irradiation with a focal boost to the tumor bed, focal re-irradiation is often considered at the time of relapse (159, 160). If these approaches demonstrate safety, strategies to combine oncolytic measles virus with local radiotherapy can be explored to enhance anti-tumor immune response and offer abscopal benefit for patients with disseminated disease.

### Perspectives on Combining Immunotherapy With Radiation – Safety and Toxicity

When evaluating the safety of immunotherapies in pediatric brain tumors, especially in combination with radiotherapy, treatment related inflammation and edema due to immune-mediated tumor cell death must be considered. As described above, clinical and radiographic pseudoprogression can occur in patients undergoing radiotherapy for the treatment of brain tumors, but the overall tolerability in the published experience with combined radiotherapy and ICI in pediatric CNS tumors is reassuring. However, as more patients are treated with these combinations, the incidence of these acute toxicities may become more apparent. Another consideration in evaluating response to combined immunotherapy and radiotherapy is the complexity of interpreting radiographic changes following therapy and potential pseudoprogression, which may make it challenging to ascertain disease progression versus treatment response.

As far as direct CNS toxicity with immune-based therapies, the greatest amount of literature is available for ICI. Anti-CTLA4 therapy is associated with auto-immune hypophysitis in 13% of patients, more so than PD-1 or PD-L1 blockade. Auto-immune thyroiditis is also reported in patient receiving ICI (161). Fortunately, endocrine dysfunction in patients affected by auto-immune hypophysitis or thyroiditis is transient and typically responds to corticosteroids. In contrast to acute neuroendocrine injury seen with ICI, radiation related neuroendocrine dysfunction is a late effect. Long term follow-up studies in patients receiving combined ICI and radiotherapy will be necessary to determine if risk of long-term neuroendocrine dysfunction is affected by this combination. Mechanistically, ICI-related hypophysitis and thyroiditis likely emerge due to on-target engagement of ICI therapy, which boosts systemic immune activation. Interestingly, analyses of adverse events in metastatic melanoma patients receiving ICI has revealed a positive correlation between development of vitiligo, an autoimmune attack of normal melanocytes, and treatment response (162). However, incidence of auto-immune injury of other organ systems did not exhibit this correlation. Hypotheses for this phenomenon include the shared immunoreactivity of anti-tumor T cells toward antigens in the normal melanocytes. It remains to be determined if similar autoimmune phenomenon will be observed in brain tumor patients receiving immunotherapy. This highlights the importance of treating patients on clinical trials with thorough adverse event monitoring can occur.

As investigations around immunotherapy for pediatric CNS tumors continues, efforts are underway to identify biomarkers that predict response to agents like ICI. However, much remains unknown about the molecular determinants of immune environment and subsequently on the potential response to immunotherapies. As summarized above, some understanding is beginning to emerge. For example, tumors harboring *TP53* mutations are likely associated with greater immune suppression, while tumors with MAPK pathway activation are associated with a more immunogenic environment. As a result, different patients likely require different levels of immunotherapy to achieve therapeutic response. This highlights the need to prospectively investigate immunotherapy on clinical trials where correlative studies, such as pre- and post-treatment biopsies, can be performed to provide hypothesis generating data on determinants of response. Such data will aid in identifying novel drug combinations that can overcome the immunosuppressive environment.

## Discussion

Given crosstalk between the mechanisms underlying various cancer therapies and the ongoing need for better therapies for many CNS tumors affecting children and young adults, it is vital to continue exploring novel combination strategies of radiation, targeted agents, and immune-based therapies (**Figure 1**). Each of these singular approaches offers potential clinical benefit to patients, but by bringing these interventions together, benefit will ideally be augmented. Enhancing our understanding of the molecular and immune drivers of pediatric CNS tumors, will lead to improved translation of novel combination therapy strategies to clinical practice. Such combination approaches will hopefully take advantage of some of the vulnerabilities described in this report and provide new, multi-modal approaches to target high-risk tumors like DMG and recurrent medulloblastoma. In exploring these approaches, potential areas of resistance, as determined by intrinsic patient or tumor characteristics, will need to be considered to ensure selection of patients with the greatest potential to receive clinical benefit. Safety and tolerability will remain of key importance as well, given that combination strategies may confer additive clinical benefit, but could come at a cost of additive toxicity. Within the pediatric context it will also be critical to establish measures that will allow researchers to collect long-term functional outcomes such as endocrine function and cognitive measures, as the impact of these new strategies on the developing brain remain poorly understood. Lastly, the importance of collecting informative biologic specimens will be necessary to provide insight into further patient stratification for combination therapies, validate hypotheses generated from preclinical work, and provide new hypotheses based on pathways of response or resistance. Such efforts will provide foundation from which we can make progress towards improved survival for patients with some of the greatest clinical need.

## Author Contributions

BQ: conceptualization; writing, original draft; writing, review and editing. CK: conceptualization; writing, review and editing. SM: conceptualization; writing, review and editing. All authors contributed to the article and approved the submitted version.

## Funding

CK’s work is supported the Kortney Rose Foundation.

## Conflict of Interest

The authors declare that the research was conducted in the absence of any commercial or financial relationships that could be construed as a potential conflict of interest.
